# Maternal mortality revisited: the application of the new ICD-MM classification system in reference to maternal deaths in Sri Lanka

**DOI:** 10.1186/1742-4755-11-17

**Published:** 2014-02-26

**Authors:** Suneth Agampodi, Kolitha Wickramage, Thilini Agampodi, Upuli Thennakoon, Nirasha Jayathilaka, Dhammika Karunarathna, Sasanka Alagiyawanna

**Affiliations:** 1 Maternal and Child Health Research Unit, Faculty of Medicine and Allied Sciences, Rajarata University of Sri Lanka, Mihintale, Sri Lanka; 2 International Organization for Migration, Colombo, Sri Lanka

## Abstract

**Background:**

The recently published WHO guidelines on *applications of ICD-10 to deaths during pregnancy, childbirth, and the puerperium* (ICD-MM) aimed at enabling a comprehensive framework for international comparison of maternal deaths, which includes maternal suicides as a direct cause of maternal deaths. At present, most developing countries do not include suicide as a maternal death.

**Methods:**

We extracted and analysed data from the maternal death surveillance system in North Central Province of Sri Lanka for the period of 2005 to 2011, in order to identify the implications of this new classification on maternal mortality estimates. All reported deaths of pregnant women and women within 12 months of termination of pregnancy were included in this study. Causes of deaths were extracted and coded using ICD-10 reclassified according to new ICD-MM for maternal deaths.

**Results:**

Of the 118 deaths analysed, the maternal death investigation system had classified 53 (44.9%) deaths as maternal deaths. These 53 maternal deaths included one deaths due to suicied, out of 21 (17.8%) suicide deaths among 118 reported deaths. Application of the new ICD-MM showed 83 maternal deaths which resulted in a 56.6% increase of number of maternal deaths in the province. Detailed analysis of all individual causes by ICD 10 codes showed that intentional self-poisoning by an exposure to pesticide (ICD code X63) as the leading cause of maternal deaths in NCP (n = 11, 13.3% of all maternal deaths) during this period. The estimated MMR in the study area based on the new classification in years 2009, 2010 and 2011 was 115, 103 and 88 per 100,000 live births respectively.

**Conclusions:**

The new classification system may have an immediate effect in raising maternal mortality thresholds, making the MDG Goal 5A more elusive for many countries. However, this new approach may ultimately lead to a more accurate understanding of maternal mortality, as well as the real number of maternal deaths attributed to suicide. This more accurate accounting has implications for policymakers andpractitioners globally as they strive to meet women's needs during pregnancy, including attention to detection and treatment for maternal depression, given its close correlation with maternal suicide.

## Background

Despite an estimated 47% decline in global maternal mortality (MM) from 350,000 in 1990 to 287,000 in 2010, around 40 countries are still having high maternal mortality ratios (MMR) of more than 300 per 100,000 live births [1]. However, data on MM from a large number of countries are still not reliable and around 21 UN member states have been reported as not having data on MM for the period 1980 to 2008 [2]. Even in countries where MM data are available, the inconsistency of reporting, poor quality and coverage of MM surveillance continues to limit effective implementation of prevention and control strategies of maternal mortality and morbidity [3]. Further, the lack of understanding on root causes of maternal deaths remains a major challenge to achieve the MDG goal 5A on “reducing maternal deaths by 75%” by 2015.

In 2012, the WHO working group on maternal mortality and morbidity classification developed the “Applications of ICD-10 to deaths during pregnancy, childbirth, and the puerperium” (ICD-MM). The primary objective of ICD-MM is stated as “to facilitate the consistent collection, analysis and interpretation of information on maternal deaths” [4]. In order to foster a common framework for international comparison of maternal deaths, the working group recommended the use of the guide in conjunction with ICD-10 in MM surveillance.

In ICD-MM, the underlying causes of maternal deaths are categorized in to nine groups, of which six are under direct maternal deaths. These six groups include pregnancies with abortive outcomes, hypertensive disorders in pregnancy, obstetric haemorrhages, pregnancy-related infections, other obstetric complications and unanticipated complications of management. The group five (other obstetric complications) includes maternal suicides as an underlying cause of direct maternal death, even if it is not possible to link or establish the diagnosis of postpartum depression or puerperal psychosis. This classification of suicide is a major deviation from the existing maternal mortality reporting. At present, most countries including Sri Lanka do not classify suicide as a cause of maternal death unless the underlying cause is verified as puerperal psychosis or postnatal depression. In developed countries where direct obstetric causes are low, suicide is included as an indirect cause of maternal deaths, but not as a direct cause [5-9]. This change of classification might have direct implications on MM estimates in countries where suicide is a major public health issue. There are no reports available on application of the new ICD-MM in global literature to identify implications of this new grouping and classification system.

Sri Lanka provides an interesting case study to see the implications of new ICD-MM. Sri Lanka is having a MMR of 33.4 per 100,000 live births [10]. And one of the few countries in Asia that has already achieved MDG goal 5A well ahead of the target period [2]. The maternal death surveillance system in Sri Lanka is well established and includes a combination of confidential inquiry like institutional investigation and verbal autopsy in field. The maternal deaths are classified at district level by a team of experts. The final decision on whether or not to include reported deaths in the national MM statistics is taken at National level. In this paper, we re-classified and analysed data from the maternal death surveillance system in one large province in Sri Lanka to identify the implications of ICD-MM paying attention to maternal suicides.

## Methods

The present study was carried out in the North Central province (NCP) of Sri Lanka. NCP is the largest province (by extent) in the country with a population of 1,259,200. We extracted data for the present study from the district maternal mortality surveillance reports from the office of regional director of health services of Anuradhapura and Polonnaruwa districts; the two districts in NCP. All reported deaths of pregnant and post partum women and women who delivered within last 12 months (late maternal deaths) were included in this study. Administrative clearance for use of secondary data was obtained from the provincial director of health services and medical officer- maternal and child health in each district. Summary reports as well as individual investigation reports were examined since 2005 from two districts of NCP. Demographic details, underlying causes, timing of death and the final classification of the death after maternal mortality review were extracted from the available reports. Since the available reports do not include ICD-10 classification, coding of underlying causes of deaths were done using available details on causes of deaths. Re-grouping of maternal deaths according to the ICD-MM was done based on documented underlying causes. Data on live births to calculate MMR was obtained from the maternal and child health statistics at the regional director of health services in Anuradhapura and Polonnaruwa districts.

## Results

For the period of 2005 to 2011, details of 118 deaths were extracted from Anuradhapura and Polonnaruwa districts. Data were incomplete for the period 2005 to 2008. The complete data set was available for the period 2009 to 2011. All available data were used for the reclassification. Only the completed dataset was used for estimating the maternal mortality ratio using new classification. These deaths included 10 (8.5%) unmarried women and 13 (11.0%) teenagers. Of the 118 deaths we included in our analysis, the maternal death investigation system had classified 53 (44.9%) deaths as maternal deaths after the national level review (Table 1).

**Table 1:** Documented classification of 118 deaths captured by the maternal mortality surveillance system of North Central Province, Sri Lanka during 2005-2011 period compared to reclassification based on ICD-MM

	**Previous classification**^**1**^		**ICD-MM classification**^**2**^	
**Maternal death category**	**n**	**%**	**n**	**%**
Direct maternal deaths	36	30.5	52	44.1
Indirect maternal deaths	17	14.4	31	26.3
Late maternal deaths	14	11.9	14	11.9
Pregnancy related deaths	50	42.4	19	16.1
Inconclusive	1	0.8	1	.8

^1^This is the classification documented in reports.

^2^Re-classification using the details in maternal mortality investigation reports.

Of the 118 reported deaths, 21 (17.8%) were due to suicide. Of these suicide deaths, one was reported as a direct maternal death associated with postpartum depression, two were reported as late maternal deaths and all others (n = 18) were classified as pregnancy related deaths (mentioned as incidental or accidental in maternal death investigation summary reports). These 18 deaths were not included in the maternal mortality statistics.

ICD-10 classification of suicide deaths (excluding the two late maternal deaths) showed deaths related to six categories and the leading cause was Intentional self-poisoning by and exposure to pesticides (Table 2).

**Table 2:** ICD 10 coding of 19 suicide deaths among pregnant and postpartum women reported from North Central Province, Sri Lanka from 2006 to 2011 (excluding two late maternal deaths due to suicide)

**ICD-10 code**	**ICD-10 Classification**	**n**	**%**^**1**^
X63	Intentional self-poisoning by and exposure to pesticides	11	13.3
X64	Intentional self-poisoning by and exposure to other and unspecified drugs, medicaments and biological substances	2	3.6
X68	Intentional self-harm by smoke, fire and flame	2	3.6
X70	Intentional self-harm by hanging, strangulation and suffocation	2	2.4
X71	Intentional self-poisoning by and exposure to other drugs acting on the autonomic nervous system	1	1.2
X76	Intentional self-harm by drowning and submersion	1	1.2

^1^Percentage calculated for total maternal deaths (83).

Reclassification of these 104 reported deaths (after excluding 14 late maternal deaths) according to the proposed ICD-MM showed an increase of maternal deaths that should be included in the maternal mortality statistics (Tables 1 and 3). Reclassification resulted in the number of maternal deaths rising from 53 to 83, a 56.6% increase (Table 3). These increase numbers were mainly due to suicide (18) and indirect cause of maternal deaths, which included infections as well as malignancies under the new classification. Remaining 20 were due to external causes. Indirect causes accounted for 29.8% of all maternal deaths (n = 31), followed by direct causes due to “other” obstetric complications (n = 23, 22.1% of maternal deaths). These other causes include 21 deaths due to suicides. Detailed analysis by ICD 10 codes of all these groups showed (data not shown) that intentional self-poisoning by an exposure to pesticide (ICD code X63) as the leading cause of maternal deaths in NCP (n = 11, 11.2% of all maternal deaths). The estimated MMR in NCP based on the new classification in years 2009, 2010 and 2011 was 115, 103 and 88 per 100,000 live births respectively.

**Table 3:** Classification of 104 maternal deaths in North Central Province, Sri Lanka from 2006 to 2011 according to ICD-MM guide (excluding late maternal deaths)

**Type**	**Group number/name**	**n**	**%**
Maternal death: direct	1. Pregnancies with abortive outcome	12	11.5
Maternal death: direct	2. Hypertensive disorders in pregnancy, childbirth, and the puerperium	5	4.8
Maternal death: direct	3. Obstetric haemorrhage	9	8.7
Maternal death: direct	4. Pregnancy-related infection	3	2.9
Maternal death: direct	5. Other obstetric complications	23	22.1
Maternal death: direct	6. Unanticipated complications of management	0	0
Maternal death: indirect	7. Non-obstetric complications	31	29.8
Maternal death: unspecified	8. Unknown/undetermined	2	1.9
Coincidental causes	9. Death during pregnancy, childbirth and the puerperium due to external causes	19	18.3

## Discussion

We present here the application of the new ICD-MM and show the dramatic effect on MM estimates due to inclusion of maternal suicides as a direct cause of maternal death using the Sri Lankan case study. Although the data use in this study was not complete as we observed during the data extraction procedure, these estimates provide good insight into the implications of ICD-MM.

Maternal suicides presents an important, yet neglected public health topic in global maternal health agenda and a major challenge in Sri Lanka’s health system. Previous studies, mainly in developed countries showed that suicide is the leading cause [5] or within the leading causes [8,11] of maternal deaths in some countries. Few developing countries also documented suicide as a leading cause among high risk groups [12]. However, it has been traditionally considered as an indirect cause [3,8,9,11,12] of maternal death and even in some developed countries, deaths due to suicide are not included in the maternal mortality investigation system.

While the pesticide poisoning is a well-known leading public health problem in Sri Lanka and links to suicide are well established [13], high level of maternal depression in this community is poorly being linked to maternal suicide. Our recent studies on maternal depression in Sri Lanka showed that the prevalence of antenatal and postnatal depression was 27.1% [14] and 16.2% [15] respectively. These data indicates that mental health in pregnancy should be a major focus in Sri Lankan maternal health agenda. Suicide prevention strategies and psychological intervention relevant to pregnancy are not a part in training curricula and in-service training packages of key primary health care workers such as the Public Health Midwife (PHM). In Sri Lanka, the current *Maternal Care Package for field health workers* published in 2012, provides no technical or administrative guidance for primary health care workers on addressing maternal mental health well-being, especially in regards to suicide prevention [16]. It is recommended in this field guide to use Edinburgh Postpartum Depression Scale (EPDS) as a screen tool to detect postpartum depression. However, the public health field staffs, including the community physicians are not given any training on screening, referral or follow-up procedures. Our experience on maternal suicide investigation clearly shows that application of EPDS is still not considered as a priority by the field staff. Screening programme might help to initiate the secondary prevention activities, but the maternal care programme needs a more focus on primary prevention. There is also lack of research in identifying the risk and protective factors and social determinants contributing to maternal suicide as done in other settings [11,17]. The focus of global maternal health agenda is primarily focus on well-known and well researched areas such as septic abortion, haemorrhage, hypertensive disorders in pregnancy and cardiac diseases complicating pregnancy, whereas maternal mental health and morbidity and mortality related to this areas is often neglected. While the WHO ICD-MM provide the initial step to fill this gap, major stakeholders of global maternal health should have more focus on filling research gaps to provide more evidence on prevention of maternal depression. Such evidence will be vital in formulating suicide prevention and case-management strategies.

In addition to the inclusion of suicide as a direct cause of maternal death, inclusion of dengue and other infections as indirect causes of maternal deaths also increases the number of maternal deaths in this analysis. In a country with dengue hyper endemic and deaths due to pneumonia is not uncommon, this will also lead to increase the MMR in country. In this analysis we observed 3 deaths due to dengue and 5 due to pneumonia.

The new WHO classification system may have an immediate effect in raising MM thresholds, making the MDG Goal 5A more elusive for many countries. In Sri Lanka, the target for 2015 was to achieve MMR of 30 per 100,000 live births. The new classification will make this target unrealistic even for a country like Sri Lanka, where maternal healthcare is considered outstanding. The new classification may also stimulate health system planners and policy makers to invest and enable psychosocial and mental health interventions within routine maternal health programs. Though challenging, the new classification approach may ultimately lead to a more positive and resilient pregnancy for many women worldwide.

## Conclusions

The proposed classification of underlying causes of maternal mortality in ICD MM will be resulted in an initial increase of maternal death rates. However, it will provide more opportunities to look in to neglected causes of maternal morbidity and mortality and to improve health of pregnant women.

## Competing interests

The authors declare that they have no competing interests.

## Authors’ contributions

SBA conceived the study. SA, TA, UT, NJ, and DK designed and carry out the study. SA involved in field work, data handling and interpretation. SBA and TA prepared the manuscript. KW involved in data acquisition, data interpretation and manuscript preparation. All authors read and approved the final manuscript.
